# Real-world characteristics, treatment experiences and corticosteroid utilisation of patients treated with tofacitinib for moderate to severe ulcerative colitis

**DOI:** 10.1186/s12876-022-02215-y

**Published:** 2022-04-09

**Authors:** Michael V. Chiorean, Jessica R. Allegretti, Puza P. Sharma, Benjamin Chastek, Leonardo Salese, Elizabeth J. Bell, Jesse Peterson-Brandt, Joseph C. Cappelleri, Xiang Guo, Nabeel Khan

**Affiliations:** 1 Swedish Medical Center, Seattle, WA USA; 2 Brigham and Women’s Hospital, Boston, MA USA; 3 Pfizer Inc, New York, NY USA; 4 Optum Life Sciences, HEOR, 11000 Optum Circle, Eden Prairie, MN 55344 USA; 5 Pfizer Inc, Collegeville, PA USA; 6 Pfizer Inc, Groton, CT USA; 7 Perelman School of Medicine, University of Pennsylvania, Philadelphia, PA USA

**Keywords:** Tofacitinib, Small molecule Janus kinase inhibitor, Tumour necrosis factor inhibitor (TNFi), Vedolizumab, Ulcerative colitis, Adherence, Oral corticosteroid

## Abstract

**Background:**

Tofacitinib is an oral, small molecule JAK inhibitor for the treatment of UC. We aimed to describe the real-world treatment experience and corticosteroid utilisation of patients treated with tofacitinib in a US claims database.

**Methods:**

Patients with a UC diagnosis who initiated tofacitinib, vedolizumab or tumour necrosis factor inhibitor (TNFi) treatment between May 2018 and July 2019 were identified from the Optum Research Database. Demographic and clinical characteristics of patients who initiated tofacitinib, vedolizumab or TNFi were described. Oral corticosteroid use prior to and following tofacitinib initiation was evaluated. Tofacitinib adherence (proportion of days covered) and continuation was assessed for 6 months following initiation. Analyses were descriptive and stratified by prior biologic use (naïve, 1 or ≥ 2; minimum of 12 months prior to tofacitinib initiation).

**Results:**

Among patients initiating tofacitinib (N = 225), mean age was 45.6 (SD 16.5) years and 50.2% were female. Of these, 43 (19.1%) patients were biologic-naïve and 182 (80.9%) had prior biologic use (92 [40.9%], 1 prior biologic; 90 [40.0%], ≥ 2 prior biologics). Among patients with 1 prior biologic, 82.6% were previously treated with a TNFi. Among patients with ≥ 2 prior biologics, 54.4% were previously treated with vedolizumab and a TNFi, 16.7% with two TNFi and 28.9% with ≥ 3 prior biologics. In the 6 months prior to tofacitinib initiation, 65.8% of patients had received oral corticosteroids (74.4%, 60.9% and 66.7% for biologic-naïve, 1 and ≥ 2 prior biologics, respectively). The proportion of patients with ongoing oral corticosteroid use 3–6 months after tofacitinib initiation decreased to 13.3% (9.3%, 18.5% and 10.0% for biologic-naïve, 1 and ≥ 2 prior biologics, respectively), and 19.6% of patients discontinued oral corticosteroid use during the 6 months after tofacitinib initiation. Overall, tofacitinib adherence, as determined by the mean proportion of days covered during the 6-month follow-up, was 0.7 (median 0.8). During the 6-month follow-up, 84.9% of patients continued tofacitinib.

**Conclusions:**

Among patients with UC initiating tofacitinib, the majority had prior biologic use. Tofacitinib adherence was high, discontinuation was low and oral corticosteroid utilisation decreased irrespective of prior biologic use. Further research with longer follow-up and a larger sample size is required.

**Supplementary Information:**

The online version contains supplementary material available at 10.1186/s12876-022-02215-y.

## Background

Ulcerative colitis (UC) is a chronic inflammatory disease of the colon, of which the aetiology is unknown [1]. UC requires life-long treatment and, despite the increasing spectrum of therapeutic options available, treatment failure is common. There is an unmet medical need to achieve optimal treatment goals of reducing utilisation and dependency on steroids for maintaining a therapeutic response and reducing colectomy rates [2].

Tofacitinib is an oral, small molecule Janus kinase inhibitor for the treatment of UC. The efficacy and safety of tofacitinib has been evaluated in an 8-week, phase II induction study (NCT00787202) [3], two 8-week, phase III induction studies (OCTAVE Induction 1 and 2, NCT01465763 and NCT01458951), a 52-week, phase III maintenance study (OCTAVE Sustain, NCT01458574) [4] and an open-label, long-term extension study (OCTAVE Open, NCT01470612) [5]. The results from these pivotal trials may influence regulatory approval, expert recommendations and evidence-based practice guidelines for the management of patients with UC [6]. Nevertheless, the limitation of the clinical trial patient population, via stringent inclusion and exclusion criteria, may not be fully representative of the patient population encountered in clinical practice, as demonstrated by a retrospective study in which only 26% of patients with UC were eligible to participate in such clinical trials [7]. Therefore, studies evaluating the efficacy and safety in the real-world setting offer a reliable, alternative source of information to guide treatment decisions for patients with UC.

To date, the effectiveness and safety of tofacitinib in patients with UC in the real-world setting have been confirmed in a number of small studies across Europe [8–12] and the US [13]. A prospective tofacitinib registry is also in development to evaluate efficacy and safety in a real-world setting (NCT03772145). Nonetheless, only limited data are available on the profile of patients with UC treated with tofacitinib in this setting. The objective of this study was to evaluate the demographic and clinical characteristics of patients with UC treated with tofacitinib, vedolizumab or tumour necrosis factor inhibitors (TNFi), and to evaluate the adherence, persistence and corticosteroid sparing efficacy in patients with UC treated with tofacitinib in the US, overall and stratified by prior biologic use.

## Methods

### Data source and study design

This was a retrospective cohort study of adjudicated claims from the Optum Research Database (a large, US-based repository of de-identified administrative claims data for more than 111 million enrolees with commercial or Medicare Advantage health plan information) for the period of 30 May 2018–31 July 2019.

### Ethical considerations

The study only used data from the Optum Research Database which have been de-identified in compliance with 45 Code of Federal Regulations 164.514(a)–(c). The de-identified data in the Optum Research Database were obtained from Covered Entities that permitted de-identification of protected health information for use in research studies conducted by Optum. In the United States, research involving human subjects is subject to the United States Department of Health and Human Services (HHS) “Common Rule”, codified at 45 C.F.R. Part 46, which includes requirements for IRB review to ensure adequate protections of those human subjects. However, in this case, the research has been conducted with de-identified Protected Health Information (PHI). The PHI has been de-identified in accordance with the HHS Privacy Rule’s requirements for de-identification codified at 45 C.F.R. § 164.514(b). Therefore, the research is not subject to the Common Rule requirements and an IRB review.

Throughout the process, patient privacy was preserved, and researchers complied strictly with all applicable Health Insurance Portability and Accountability Act data management rules and the 1964 Helsinki Declaration and its later amendments or comparable ethical standards. Patient consent was not required for this retrospective study.

### Study population

From the Optum Research Database [14], patients with UC were identified using the following selection criteria: patients were ≥ 18 years of age on the index date and had a diagnosis of UC, as defined by the International Classification of Diseases, 9th or 10th Revision 556 and/or K51 codes during the 12-month baseline period. Patients with only UC diagnostic codes related to diagnostic tests were excluded. Patients with a diagnosis of UC and a claim code for a rheumatological condition were not excluded. The index date was defined as the date of the first claim for index therapy (tofacitinib, vedolizumab or TNFi). The index date was assigned hierarchically and the index date for patients initiating tofacitinib was set as the first date of the claim for tofacitinib and for patients with biologic use, the index date was the first claim date for a biologic. The 12-month baseline period was defined based on patients’ continuous health plan enrolment with medical and pharmacy benefits in the 12 months prior to the index date. For patients with continuous health plan enrolment of more than 12 months prior to the index date, a variable-length baseline of up to 6.6 years prior to initiation of index therapy was examined in order to identify prior biologic use. For each index therapy (tofacitinib, vedolizumab or each specific TNFi), patients were required to be new to the index therapy but could have received a different index therapy during the variable-length (minimum 12-month) pre-index baseline period; for example, a patient initiating tofacitinib could have received vedolizumab but not tofacitinib during the variable-length pre-index baseline period. Patients were followed-up for 6 months following the initiation of index therapy. A sensitivity analysis with 12 months of follow-up was conducted for patients with available data.

### Study cohorts

The tofacitinib study population was stratified into three cohorts, based on exposure to biologic therapy during the variable-length baseline period: biologic-naïve, 1 prior biologic and ≥ 2 prior biologics. Biologics approved in the US for the treatment of moderate to severe UC at the time of the study were included (TNFi [adalimumab, infliximab, golimumab] and vedolizumab). For the purposes of this analysis, each type of TNFi was viewed as a unique biologic. The approval date of ustekinumab for ulcerative colitis (October 2019) precluded its inclusion in the study.

### Study measures

Patient demographics and clinical characteristics, including age, sex, comorbidities, geographical region, baseline inflammatory conditions and prior medical treatment of patients who initiated tofacitinib, vedolizumab and TNFi, were collected. Prior medical treatment was identified from claims data using National Drug Codes (NDC) and Healthcare Common Procedure Coding System (HCPCS) codes. Comorbid conditions were defined using the Clinical Classifications Software in accordance with the Agency for Healthcare Research and Quality (AHRQ) standards [15, 16]. Patients’ medical claims during the 12-month baseline period were used to calculate a Quan-Charlson comorbidity score [17, 18]. The Quan-Charlson comorbidity score updated the Charlson index, enhancing its use with administrative claims data. Comorbidity categories are weights (from 1 to 6), the sum of which is the overall score for a patient, where zero indicates the absence of comorbidities and a higher score indicates higher severity [18].

Data on 5-aminosalicylates (5-ASA), immunomodulators and oral corticosteroid (excluding budesonide) use in the 6 months prior to the index date and in the 6-month follow-up period were collected. Patients with a new oral corticosteroid prescription were defined as those who had a prescription during Months 4–6 of follow-up, but did not have a prescription during Months 1–3. Patients with an ongoing oral corticosteroid prescription were defined as those who had a prescription during Months 1–3 and Months 4–6 during the 6-month follow-up period. Patients who discontinued oral corticosteroids were defined as those who had a prescription during Months 1–3, but did not have a prescription during Months 4–6.

Tofacitinib adherence and persistence were measured using claims data for the index therapy, inclusive of fills, on the index date [19]. Adherence to tofacitinib was assessed with the proportion of days covered method. The proportion of days covered was calculated by dividing the number of days on which medication was available (based on filled prescriptions) by the number of days between the earliest prescription claim in the observation period through the end of the observation period. Discontinuation of tofacitinib was defined as a gap in therapy of 60 or more days. The discontinuation date was defined as the time when the days’ supply from the last filled prescription ran out prior to the gap in therapy. Persistence with tofacitinib, defined as the time to discontinuation of tofacitinib, was calculated. Persistence was calculated with data up to 60 days before the end of follow-up due to the required 60-day gap.

### Statistical analysis

The baseline characteristics of the three index therapy cohorts were compared, and all other analyses were restricted to patients who initiated tofacitinib. All study variables were analysed descriptively and for patients initiating tofacitinib, stratified by prior biologic history during the baseline period. Mean, standard deviation (SD), median and quartiles were calculated for continuous measures. Counts and percentages were calculated for categorical variables [20]. All analyses were conducted with statistical software SAS 9.4 (SAS Institute, Cary, NC, USA). Kaplan–Meier time-to-event analyses were performed to estimate time to treatment discontinuation [21].

## Results

### Patients and baseline characteristics by index therapy

**Fig. 1:**
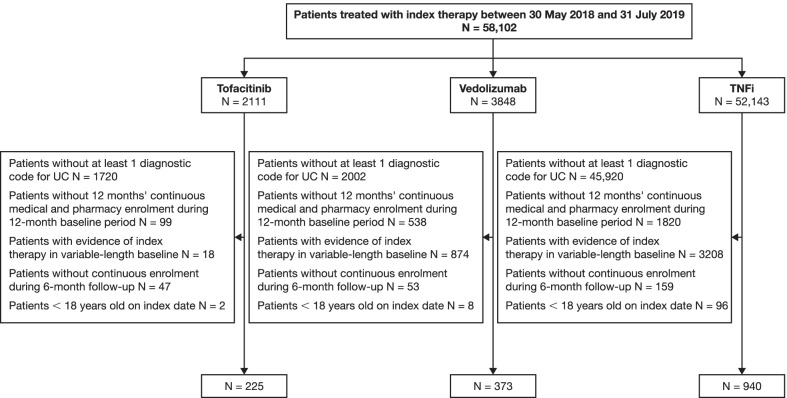
Patient disposition. Patients with only UC diagnostic codes related to diagnostic tests were excluded. N, number of patients meeting the inclusion/exclusion criteria; TNFi, tumour necrosis factor inhibitor; UC, ulcerative colitis

A total of 1538 patients with UC met the study inclusion criteria, of whom 225 (14.6%) had initiated tofacitinib, 373 (24.3%) had initiated vedolizumab and 940 (61.1%) had initiated a TNFi (Fig. 1).

Baseline demographics and characteristics of patients who had initiated tofacitinib, vedolizumab or a TNFi were generally similar, with the exception of biologic treatment history (Table 1). The majority of patients who initiated vedolizumab (82.3%) or TNFi (93.7%) were biologic-naïve compared with patients who initiated tofacitinib (19.1%), and a greater proportion of patients who initiated tofacitinib had previously received ≥ 2 biologics compared with patients who initiated vedolizumab or TNFi, making any further comparisons of outcomes between these groups challenging (Table 1). Compared with patients who initiated tofacitinib or TNFi, a higher proportion of patients who initiated vedolizumab were ≥ 65 years of age (Table 1). The proportion of patients with oral corticosteroid use in the 12-month baseline period was similar in patients who initiated tofacitinib, vedolizumab or a TNFi.

**Table 1:** Baseline demographic and clinical characteristics of patients with UC initiating tofacitinib, vedolizumab or a TNFi

Demographics	Tofacitinib (N = 225)	Vedolizumab (N = 373)	TNFi (N = 940)
Duration of variable-length baseline period (years), mean (SD)	3.4 (1.8)	3.5 (1.8)	3.5 (1.8)
Age at index date (years), mean (SD)	45.6 (16.5)	48.7 (18.0)	45.8 (17.1)
Gender, n (%)			
Female	113 (50.2)	188 (50.4)	466 (49.6)
Male	112 (49.8)	185 (49.6)	474 (50.4)
Biologic treatment history, n (%)			
Biologic-naïve	43 (19.1)	307 (82.3)	881 (93.7)
1 prior biologic	92 (40.9)	49 (13.1)	49 (5.2)
≥ 2 prior biologics	90 (40.0)	17 (4.6)	10 (1.1)
Oral corticosteroid use, n (%)^a^	173 (76.9)	254 (68.1)	693 (73.7)
Geographic region, n (%)			
Northeast	26 (11.6)	46 (12.3)	102 (10.9)
Midwest	49 (21.8)	108 (29.0)	263 (28.0)
South	97 (43.1)	162 (43.4)	427 (45.4)
West	53 (23.6)	57 (15.3)	148 (15.7)
12-month baseline Quan-Charlson comorbidity score, mean (SD)	0.7 (1.3)	0.8 (1.5)	0.8 (1.3)
12-month baseline Quan-Charlson comorbidity score, n (%)			
0	152 (67.6)	233 (62.5)	602 (64.0)
1	34 (15.1)	57 (15.3)	154 (16.4)
2	23 (10.2)	48 (12.9)	97 (10.3)
3	8 (3.6)	13 (3.5)	45 (4.8)
4	3 (1.3)	9 (2.4)	22 (2.3)
≥ 5	5 (2.2)	13 (3.5)	20 (2.1)

n, number of patients in the specified category; N, number of patients in the treatment group; SD, standard deviation; TNFi, tumour necrosis factor inhibitor; UC, ulcerative colitis

^a^During the 12-month baseline period

### Patients and baseline characteristics in the tofacitinib study population, overall and stratified by prior biologic use

Baseline demographics and clinical characteristics of patients who initiated tofacitinib, overall and stratified by prior biologic use, are shown in Table 2. Of the 225 patients with UC receiving tofacitinib, 43 (19.1%) were biologic-naïve, 92 (40.9%) had initiated tofacitinib after previous treatment with 1 biologic and 90 (40.0%) had initiated tofacitinib after previous treatment with ≥ 2 biologics, during the baseline period. Among patients who had previously received 1 biologic, 82.6% (76/92) had prior treatment with a TNFi and 17.4% (16/92) had prior treatment with vedolizumab. In patients with prior treatment with ≥ 2 biologics, 54.4% (49/90) were treated with vedolizumab and a TNFi, 16.7% (15/90) had been treated with two TNFi and 28.9% (26/90) had received ≥ 3 biologics (Table 2). When stratified by prior biologic use, the proportion of female patients and mean age of patients was similar across the three cohorts (Table 2). Most (88.4%) patients initiated tofacitinib treatment at 20 mg/day, while 11.6% of patients initiated tofacitinib at 10 mg/day.

**Table 2:** Baseline demographic and clinical characteristics of tofacitinib-treated patients, overall and by prior biologic history

Demographics	Overall	Biologic-naïve	1 prior biologic	≥ 2 prior biologics
	N = 225	N = 43	N = 92	N = 90
Duration of variable-length baseline period (years), mean (SD)	3.4 (1.8)	3.4 (1.9)	3.0 (1.8)	3.9 (1.8)
Age at index date (years), mean (SD)	45.6 (16.5)	46.5 (17.1)	45.5 (15.4)	45.2 (17.4)
Gender, n (%)				
Female	113 (50.2)	22 (51.2)	49 (53.3)	42 (46.7)
Male	112 (49.8)	21 (48.8)	43 (46.7)	48 (53.3)
12-month baseline Charlson comorbidity score, mean (SD)	0.7 (1.3)	0.5 (1.0)	0.7 (1.5)	0.7 (1.2)
Prior biologic use, n (%)				
Adalimumab	101 (44.9)	0 (0.0)	44 (47.8)	57 (63.3)
Infliximab	87 (38.7)	0 (0.0)	28 (30.4)	59 (65.6)
Golimumab	27 (12.0)	0 (0.0)	4 (4.4)	23 (25.6)
Vedolizumab	90 (40.0)	0 (0.0)	16 (17.4)	74 (82.2)
Number of unique biologics, n (%)				
0	43 (19.1)	43 (100.0)	NA	NA
1	92 (40.9)	NA	92 (100.0)	NA
2	64 (28.4)	NA	NA	64 (71.1)
Vedolizumab + TNFi	49 (21.8)	NA	NA	49 (54.4)
TNFi + TNFi	15 (6.7)	NA	NA	15 (16.7)
≥ 3	26 (11.6)	NA	NA	26 (28.9)

n, number of patients in the specified category; N, number of patients in the treatment group; NA, not applicable; SD, standard deviation; TNFi, tumour necrosis factor inhibitor

Among patients with UC who initiated tofacitinib, 44.4% (100/225) of patients had an extraintestinal manifestation during the 12-month baseline period. Among the extraintestinal manifestations reported, arthralgia was the most common, with the condition identified in 37.3% (84/225) of patients overall.

### Other medication uses in the tofacitinib study population

In the 6 months prior to tofacitinib initiation, 49.8% (112/225) of patients were treated with 5-ASA, 30.2% (68/225) were treated with an immunomodulator and 65.8% (148/225) were treated with oral corticosteroids (Table 3). During the 6 months after tofacitinib initiation, 31.1% (70/225) of patients continued to use 5-ASA, whereas the proportion of patients treated with an immunomodulator decreased to 9.3% (21/225), and 41.3% (93/225) of patients had oral corticosteroid use (Table 3). Overall, the mean daily dose of oral corticosteroids decreased in the 6 months after tofacitinib initiation (Table 3).

**Table 3:** Other medication use in the 6 months prior to and after tofacitinib initiation

	Overall		Biologic-naïve		1 prior biologic		≥ 2 prior biologics	
	N = 225		N = 43		N = 92		N = 90	
	6-month baseline	6-month follow-up	6-month baseline	6-month follow-up	6-month baseline	6-month follow-up	6-month baseline	6-month follow-up
5-ASA, n (%)	112 (49.8)	70 (31.1)	25 (58.1)	16 (37.2)	49 (53.3)	30 (32.6)	38 (42.2)	24 (26.7)
Immunomodulator, n (%)	68 (30.2)	21 (9.3)	3 (7.0)	4 (9.3)	31 (33.7)	14 (15.2)	34 (37.8)	3 (3.3)
Oral corticosteroids, n (%)^a^	148 (65.8)	93 (41.3)	32 (74.4)	10 (23.3)	56 (60.9)	39 (42.4)	60 (66.7)	44 (48.9)
Daily dose, mg/day, mean (SD)^b^	31.5 (16.2)	28.2 (13.5)	30.1 (18.2)	22.5 (17.9)	33.6 (19.7)	26.6 (14.1)	30.4 (10.6)	30.9 (11.3)

Baseline was defined as the 6 months prior to index date. Follow-up period was 6 months following the index date

5-ASA, 5-aminosalicylates; n, number of patients in the specified category; N, number of patients in the treatment group; TNFi, tumour necrosis factor inhibitor

^a^Based on pharmacy claims only

^b^Prednisone equivalent

**Fig. 2:**
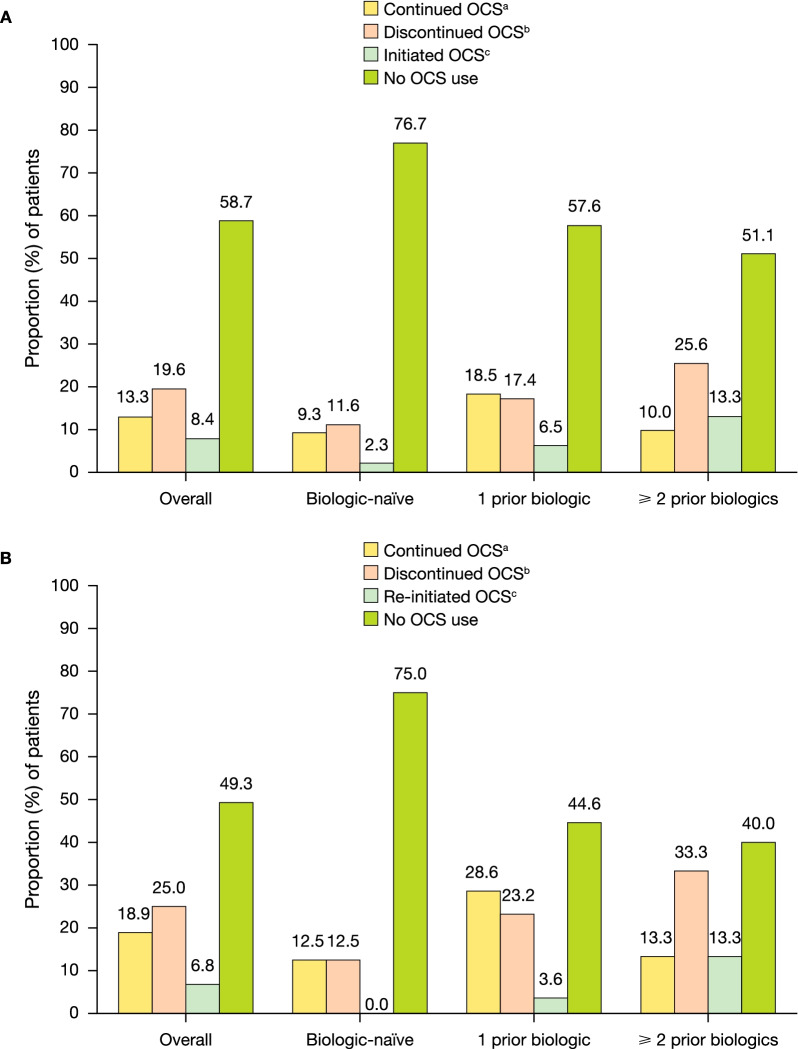
Oral corticosteroid use following tofacitinib initiation in (**a**) all patients and (**b**) in patients with oral corticosteroid use during 6-month baseline period, overall and by prior biologic history. Follow-up period was 6 months following the index date. OCS, oral corticosteroids. ^a^Patients with a prescription for OCS during Months 1–3 and Months 4–6 of follow-up. ^b^Patients with a prescription for OCS during Months 1–3 who did not have a prescription for OCS during Months 4–6 of follow-up. ^c^Patients with a prescription for OCS during Months 4–6 of follow-up who did not have a prescription for OCS during Months 1–3

In the 6 months following tofacitinib initiation, 58.7% (132/225) of patients required no oral corticosteroid use, 13.3% (30/225) of patients continued oral corticosteroids and 19.6% (44/225) of patients discontinued oral corticosteroids (Fig. 2a). During Months 4–6 of follow-up, 8.4% (19/225) of patients initiated oral corticosteroids.

During the 6-month baseline period, 148 patients had oral corticosteroid use. Of these, during the 6-month follow-up period after tofacitinib initiation, 49.3% (73/148) of patients had no oral corticosteroid use, 18.9% (28/148) of patients continued oral corticosteroids, 25.0% (37/148) of patients discontinued oral corticosteroids and 38% (10/148) of patients re-initiated corticosteroids (Fig. 2b). Overall, among patients with oral corticosteroid use during the 6-month baseline period, 74.3% (110/148) of patients did not have oral corticosteroid use during Months 4–6 of follow-up (87.5% [28/32] of biologic naïve patients; 67.9% [38/56] of patients with 1 prior biologic; 73.3% [44/60] of patients with ≥ 2 prior biologics). 

Oral corticosteroid use during the 6 months following tofacitinib initiation was consistent with results from a small cohort of patients in our study (N = 91) with 12 months of follow-up data available (sensitivity analysis). In this cohort, among patients with oral corticosteroid use during Months 1–3 of follow-up, over half had discontinued oral corticosteroids by month 12 (Additional file 1: Table S1).

### Adherence to and discontinuation of tofacitinib

During the 6-month follow-up period, the mean and median proportion of days covered for tofacitinib were similar across the three prior biologic cohorts (Table 4).

**Table 4:** Proportion of days covered and discontinuation of tofacitinib during follow-up, by prior biologic history

	Overall	Biologic-naïve	1 prior biologic	≥ 2 prior biologic
	N = 225	N = 43	N = 92	N = 90
Proportion of days covered during 6-month follow-up				
Mean (SD)	0.7 (0.3)	0.7 (0.3)	0.7 (0.3)	0.7 (0.3)
Median (IQR)	0.8 (0.5, 1.0)	0.8 (0.5, 1.0)	0.9 (0.5, 1.0)	0.8 (0.5, 1.0)
Discontinuation during 4 months of follow-up,^a^ n (%)	34 (15.1)	9 (20.9)	15 (16.3)	10 (11.1)
Time to discontinuation during 4 months of follow-up,^a^ days				
Mean (SD)	42.4 (15.0)	43.3 (15.8)	42.0 (15.2)	42.0 (15.5)
Median (IQR)	30.0 (30.0, 60.0)	30.0 (30.0, 60.0)	30.0 (30.0, 60.0)	30.0 (30.0, 60.0)

Sensitivity analysis (12 months of follow-up)	N = 91	N = 16	N = 31	N = 44
Proportion of days covered during 12-month follow-up				
Mean (SD)	0.7 (0.3)	0.8 (0.2)	0.7 (0.3)	0.6 (0.3)
Median (IQR)	0.8 (0.4, 1.0)	0.8 (0.6, 0.9)	0.8 (0.5, 1.0)	0.6 (0.3, 1.0)
Discontinuation during 10 months of follow-up,^a^ n (%)	36 (39.6)	4 (25.0)	10 (32.3)	22 (50.0)
Time to discontinuation during 10 months of follow-up,^a^ days				
Mean (SD)	120.0 (67.8)	177.8 (61.7)	131.1 (72.0)	104.4 (62.8)
Median (IQR)	99.5 (63.5, 180.0)	198.0 (135.0, 220.5)	105.5 (90.0, 180.0)	94.5 (60.0, 150.0)

The ideal proportion of days covered is 1.0

IQR, interquartile range; SD, standard deviation

^a^Patients were not considered at risk of discontinuing therapy in the last 60 days of their follow-up because of the required 60-day gap to define discontinuation. Therefore, discontinuation was only calculated up to 60 days before the end of the follow-up period

**Fig. 3:**
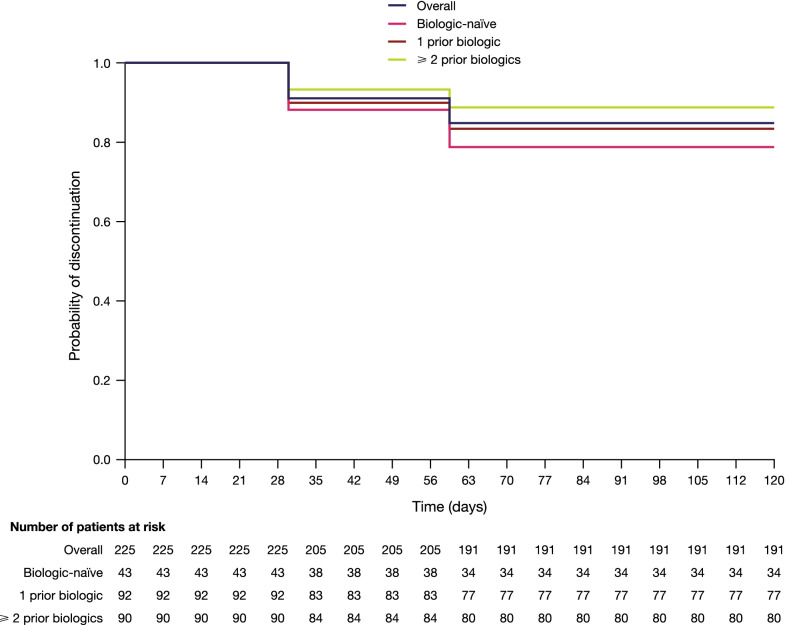
Kaplan–Meier curve for time to treatment discontinuation among patients initiating tofacitinib, by prior biologic history

As shown in Fig. 3, 84.9% (191/225) of patients were still receiving tofacitinib at 4 months. Among those who were biologic-naïve, 79.1% (34/43) continued tofacitinib, compared with 83.7% (77/92) of patients with 1 prior biologic and 88.9% (80/90) of patients with ≥ 2 prior biologics. The mean time to discontinuation of tofacitinib was 42.4 days; 43.3 days in the biologic-naïve, and 42.0 days in patients with either 1 or ≥ 2 prior biologics (Table 4). The results were consistent with results from a small cohort of patients with a follow-up of 12 months, as shown in Table 4.

## Discussion

To our knowledge, this is the first real-world study of tofacitinib in patients with UC using administrative claims data consisting of a mostly active, working age population in the US. Previously, experience of tofacitinib in patients with UC had been reported in randomised clinical trials [3, 4, 22] and real-world studies [8–13, 23], all of which have focused on effectiveness and safety of tofacitinib. However, real-world reports were of relatively smaller scope or single-centre based, compared with the current study.

In this analysis, patients were stratified into distinct cohorts based on their exposure to prior biologic agents in the pre-index period. A large proportion of patients treated with tofacitinib had received prior biologic treatment, whereas the majority of patients who initiated vedolizumab or TNFi were biologic-naïve. Furthermore, approximately half of tofacitinib-treated patients with prior treatment had received ≥ 2 biologics. Among patients who had received ≥ 2 biologics prior to initiating tofacitinib, more than half had prior treatment with vedolizumab and a TNFi, and approximately one-quarter had prior treatment with ≥ 3 biologics. This relatively high rate of biologic usage suggests that patients initiating tofacitinib in this cohort had more difficult to treat or severe disease, compared with patients initiating vedolizumab or TNFi, as has been reported in other studies on this topic [8, 11]. Despite that, following initiation of tofacitinib treatment, adherence was high and oral corticosteroid use declined, regardless of patients’ treatment history.

Patients included in this study had characteristics consistent with a more severe disease course, such as the presence of extraintestinal manifestations and frequent use of corticosteroids [6, 24]. In our population, 65.8% of patients were receiving oral corticosteroids during the 6 months prior to initiating tofacitinib treatment. Achieving and maintaining corticosteroid-free remission is an important treatment goal in patients with UC [25] and, although rates of remission were not reported in this study, we found that oral corticosteroid use decreased after tofacitinib initiation and was consistent regardless of prior biologic history.

Adherence to tofacitinib was high and discontinuation rates were low in the 6 months following tofacitinib initiation. The treatment adherence and discontinuation findings noted here are comparable with the findings from two previous real-world studies [8, 11]. A UK-based multicentre study of 134 patients with UC reported the probability of remaining on tofacitinib treatment after 8, 16 and 26 weeks to be 91%, 84% and 71%, respectively [11]. Additionally, the authors reported that prior biologic use had no evident influence on persistence with tofacitinib treatment [11]. In a Dutch study of 123 patients with UC (95% and 62% of whom had prior TNFi and vedolizumab therapy, respectively), 60% of patients treated with tofacitinib remained on tofacitinib treatment after 24 weeks of follow-up [8]. In contrast to the low discontinuation rate reported here, a study conducted in Spain of 113 patients with UC treated with tofacitinib following prior exposure to biologics (TNFi, vedolizumab or ustekinumab) showed that 40% of the patients discontinued over time, with cumulative discontinuation rates of 34% and 46% at 24 and 52 weeks, respectively [9].

Our study has some limitations. The results from this study are based on a relatively short 6-month follow-up period with information up to 12 months of follow-up being available for a small subgroup of patients. Longer follow-up studies could provide further insight into these outcomes. Other limitations inherent to the use of administrative claims data include the lack of information on UC disease duration, extent and severity, clinical response and remission status, as well as the incidence of treatment-emergent side effects. In addition, claims data do not provide insight into dose adjustments or reasons for treatment discontinuation. Approximately 11% of patients initiated tofacitinib at 10 mg/day, the recommended dose for rheumatoid arthritis and psoriatic arthritis [26, 27]; therefore, it is possible that some of the patients included in this analysis received tofacitinib for non-UC diagnoses. The index date was assigned hierarchically with the index date for patients, with evidence of tofacitinib treatment set first, and, therefore, patients who initiated tofacitinib may be more likely to have a history of biologics than if the population had been a random sample. The difference in duration and relatively short minimum length of variable-length baseline between the three prior biologic history cohorts could imply possible misclassification of the history of biologic use; however, our results suggest, overall, no difference in outcomes based on the number of biologic agents used prior to starting tofacitinib. The analysis presented here did not include ustekinumab, which was not approved for use in patients with UC at the time of this study, in the inclusion or exclusion criteria; therefore, it is possible that some patients may have been prescribed ustekinumab for non-UC diagnoses during the baseline period and this would not be reflected in the baseline biologic use.

In summary, in a real-world cohort of patients with UC, we found that patients initiating tofacitinib were more likely to have previously received other biologics compared with patients initiating vedolizumab or TNFi. There was substantial corticosteroid sparing after tofacitinib initiation and tofacitinib adherence was high regardless of prior biologic exposure. These findings provide insights into the real-world patient characteristics and experience of patients with UC receiving tofacitinib, which could be valuable to patients and healthcare providers.

## Supplementary Information

**Additional file 1: Table S1**. Sensitivity analysis of oral corticosteroid use in the 12 months following tofacitinib initiation, by prior biologic history

## Data Availability

Upon request, and subject to review, Pfizer will provide the data that support the findings of this study. Subject to certain criteria, conditions and exceptions, Pfizer may also provide access to the related individual de-identified participant data. See https://www.pfizer.com/science/clinical-trials/trial-data-and-results for more information. The data underlying the results presented in the study include administrative medical and pharmacy claims from data available from Optum and cannot be broadly disclosed or made publicly available at this time. The disclosure of this data to third-party clients assumes certain data security and privacy protocols are in place and that the third-party client has executed a standard license agreement which includes restrictive covenants governing the use of the data. Please see https://www.optum.com/content/dam/optum/resources/productSheets/Clinformatics_for_Data_Mart.pdf for more information about licensing these data from Optum.

## Abbreviations

5-ASA: 5-aminosalicylates
AHRQ: Agency for Healthcare Research and Quality
HCPCS: Healthcare Common Procedure Coding System
IQR: Interquartile range
JAK: Janus kinase
NA: Not applicable
NDC: National Drug Codes
OCS: Oral corticosteroid
SD: Standard deviation
TNFi: Tumour necrosis factor inhibitor
UC: Ulcerative colitis
