# Correction to: Real-world characteristics, treatment experiences and corticosteroid utilisation of patients treated with tofacitinib for moderate to severe ulcerative colitis

**DOI:** 10.1186/s12876-022-02298-7

**Published:** 2022-07-26

**Authors:** Michael V. Chiorean, Jessica R. Allegretti, Puza P. Sharma, Benjamin Chastek, Leonardo Salese, Elizabeth J. Bell, Jesse Peterson-Brandt, Joseph C. Cappelleri, Xiang Guo, Nabeel Khan

**Affiliations:** 1 Swedish Medical Center, Seattle, WA USA; 2 Brigham and Women’s Hospital, Boston, MA USA; 3 Pfizer Inc, New York, NY USA; 4 Optum Life Sciences, HEOR, 11000 Optum Circle, Eden Prairie, MN 55344 USA; 5 Pfizer Inc, Collegeville, PA USA; 6 Pfizer Inc, Groton, CT USA; 7 Perelman School of Medicine, University of Pennsylvania, Philadelphia, PA USA

## Correction to: BMC Gastroenterology (2022) 22:177 10.1186/s12876-022-02215-y

After publication of this article [1], it was reported that the copyright holder for this article was incorrectly given as “The Author(s)”, but should have been “Pfizer Inc. and The Author(s)”.

The original article [1] has been updated.
