# Supplementary material for: Real-world characteristics, treatment experiences and corticosteroid utilisation of patients treated with tofacitinib for moderate to severe ulcerative colitis
Source: BMC Gastroenterol. 2022 Apr 9;22:177. doi: 10.1186/s12876-022-02215-y (PMC8994921; doi:10.1186/s12876-022-02215-y)
Supplement: Supplementary file 1 — Additional file 1: Table S1. Sensitivity analysis of oral corticosteroid use in the 12 months following tofacitinib initiation, by prior biologic history [file 12876_2022_2215_MOESM1_ESM.pdf]

Supplementary Information

Supplementary Table 1. Sensitivity analysis of oral corticosteroid use in the 12 months following tofacitinib initiation, by prior biologic history

	**Biologic-naïve N = 16**	**1 prior biologic N = 31**	**≥ 2 prior biologics N = 44**
OCS use during Months 1–3 of follow-up, n (%)	4 (25.0)	13 (41.9)	17 (38.6)
Patients with OCS use during Months 1–3 who discontinued OCS,[a] n (%)	2 (50.0)	8 (61.5)	13 (76.5)
No OCS use during Months 9–12 of follow-up, n (%)	13 (81.3)	23 (74.2)	36 (81.8)
No OCS use during Months 6–12 of follow-up, n (%)	13 (81.3)	19 (61.3)	32 (72.7)

n, number of patients in the specified category; N, number of patients in the treatment group; OCS, oral corticosteroids

[a] Patients with a prescription for OCS during Months 1–3 of follow-up who did not have a prescription for OCS during Months 9–12 of follow-up; denominator is the number of patients with OCS use during Months 1–3 of follow-up
